# Stable Isolation of Phycocyanin from *Spirulina platensis* Associated with High-Pressure Extraction Process

**DOI:** 10.3390/ijms14011778

**Published:** 2013-01-16

**Authors:** Yong Chang Seo, Woo Seok Choi, Jong Ho Park, Jin Oh Park, Kyung-Hwan Jung, Hyeon Yong Lee

**Affiliations:** 1 Department of Medical Biomaterials Engineering, Kangwon National University, Chuncheon 200-701, Korea; E-Mails: yongchang2da@kangwon.ac.kr (Y.C.S.); wooseokchoi@kangwon.ac.kr (W.S.C.); 2 DAEBONG LS. Ltd., 122-9, Gojan-dong, Namdong-gu, Incheon 405-820, Korea; E-Mails: jh3park@daebongls.co.kr (J.H.P.); pjoh0303@daebongls.co.kr (J.O.P.); 3 Department of Biotechnology, Korea National University of Transportation, Chungcheongbuk-do 368-701, Korea; E-Mail: khjung@cjnu.ac.kr; 4 Department of Teaics, Seowon University, Cheongju, Chungbuk 361-742, Korea

**Keywords:** *Spirulina platensis*, phycocyanin, high-pressure extraction process

## Abstract

A method for stably purifying a functional dye, phycocyanin from *Spirulina platensis* was developed by a hexane extraction process combined with high pressure. This was necessary because this dye is known to be very unstable during normal extraction processes. The purification yield of this method was estimated as 10.2%, whose value is 3%–5% higher than is the case from another conventional separation method using phosphate buffer. The isolated phycocyanin from this process also showed the highest purity of 0.909 based on absorbance of 2.104 at 280 nm and 1.912 at 620 nm. Two subunits of phycocyanin namely α-phycocyanin (18.4 kDa) and β-phycocyanin (21.3 kDa) were found to remain from the original mixtures after being extracted, based on SDS-PAGE analysis, clearly demonstrating that this process can stably extract phycocyanin and is not affected by extraction solvent, temperature, *etc*. The stability of the extracted phycocyanin was also confirmed by comparing its DPPH (α,α-diphenyl-β-picrylhydrazyl) scavenging activity, showing 83% removal of oxygen free radicals. This activity was about 15% higher than that of commercially available standard phycocyanin, which implies that the combined extraction method can yield relatively intact chromoprotein through absence of degradation. The results were achieved because the low temperature and high pressure extraction effectively disrupted the cell membrane of *Spirulina platensis* and degraded less the polypeptide subunits of phycocyanin (which is a temperature/pH-sensitive chromoprotein) as well as increasing the extraction yield.

## 1. Introduction

Most microalgae found in marine and fresh water are green algae or blue-green algae. These algae use solar energy, carbon dioxide, and minerals within the water to grow, and their growth rate is very high [1]. Additionally, microalgae can produce various products by photosynthesis during their growth. *Spirulina platensis*, one of the blue-green algae among microalgae, is larger than other species of microalgae and is easily digested and absorbed in the human body because its cell membrane does not contain cellulose. *Spirulina platensis* contains a relatively low nucleic acid content; is composed of 55%–70% protein, 6%–9% fat, and 15%–20% carbohydrate, and is rich in minerals, vitamins, fibers, and pigments [2]. *Spirulina platensis* is an alkaliphilic Halobacteria that lives in tropical and subtropical lakes in Africa and in Central and South America. It has many unique chromoproteins known as phycobiliproteins [3]. In some countries, phycobiliprotein, a fluorescent chromoprotein, has been used as an additive in foods, cosmetic products, and medical diagnostic reagents [4].

The types of phycobiliprotein include red phycoerythrin and blue phycocyanin, which are composed of distinct α and β polypeptide subunits [5]. Phycocyanin, a blue natural pigment, is purported to be of high value, as it has been reported to have anti-aging, antioxidant, and anti-inflammatory activities as well as to suppress cancer metastases [6–9]. However, phycocyanin is very sensitive to temperature and pH changes in the environment because of its polypeptide subunits. Thus, the current method for isolating phycocyanin from phycobiliprotein in *Spirulina platensis* has limitations, such as inactivation by denaturation and disruption of phycocyanin, a long isolation time, and a high cost [10]. Accordingly, in this study, we applied a more effective separation method in terms of time and cost than the conventional separation method; we were able to isolate phycocyanin from *Spirulina platensis* in a short time at low temperature using a high-pressure process, which resulted in a high yield and less damage by denaturation and disruption of the components.

The high-pressure process was reported to make the cell membrane disassemble irreversibly due to the separation of weak bonds such as hydrogen bonds, coupling, and van der Waals bonds and to induce morphological and structural changes in macromolecules, while small molecules like amino acids and vitamins are known to suffer to a lesser extent [11]. Low-temperature and high-pressure treatments have the advantage of isolating the chromoprotein phycocyanin from *Spirulina platensis* with greater stability and at a higher level of purity. Currently, with the higher living standards and longer life expectancy due to the advancements in science and economic development, people have increasing interest in maintaining their health and youth. Skin care, in particular, has been the focus of much attention. For these reasons, the cosmetic business has been growing rapidly, and research in cosmetics and beauty is actively being conducted. Accordingly, in this study, we examined the possibility of phycocyanin as a new material in the cosmetic and beauty industry by establishing an isolation process for phycocyanin that is contained in *Spirulina platensis* using a high-pressure process and by verifying its reported antioxidant capabilities.

## 2. Results and Discussion

### 2.1. The Yield of Phycocyanin Isolated Using the High-Pressure Process

Table 1 shows the yields of phycocyanin isolated from *Spirulina platensis*. The yield using the conventional separation method was 7.2%, and the yield using the hexane separation method without the high-pressure process was 8.4%. This difference in yield was attributed to the lower phycocyanin loss due to the use of fewer chemical components and the simpler separation process. Moreover, we found that the yield of the isolated phycocyanin using both the high-pressure process and the hexane separation method increased up to 10.2%. This increase is attributable to the increased elution of biological active materials from *Spirulina platensis* as a result of the high pressure destroying the cell membrane. Furthermore, although the cell membrane of *Spirulina platensis* was destroyed by the high pressure, the temperature-sensitive polypeptide subunits (α and β) that make up phycocyanin were not damaged, thus increasing the yield [12].

### 2.2. The Purity of Phycocyanin Isolated Using the High-Pressure Process

We then compared the purities of the isolated phycocyanins obtained using the three different methods, the high-pressure process and hexane separation method, the hexane separation method alone, and the conventional separation method, by measuring the absorbances of these isolated phycocyanins at 620 nm and 280 nm and comparing, as shown in Table 2. The results showed that the purity of the phycocyanin isolated using the conventional separation method (A) was 0.884 and that the purity of the isolated phycocyanin using the hexane separation method without the high-pressure process (C) was 0.882, which was similar to (A). Compared to the results from the two processes of (A) and (C), the data from the combined process (B) showed both a better extraction yield and a higher purity. These data suggest that this high pressure extraction can maintain optimium stability of phycocyanin during the purification process due to less denaturation and broken bonds of the proteins in the complex structure. Also, the purity of the phycocyanin isolated using the hexane separation method was calculated as 0.881 using the absorbance values of 2.125 at 280 nm and 1.873 at 620 nm. The phycocyanin isolated using the high-pressure process and hexane separation method had a purity of 0.909 based on absorbances of 2.104 at 280 nm and 1.912 at 620 nm. Thus, the purity of the phycocyanin isolated using the hexane separation method and high-pressure process was slightly better than that of the phycocyanin standard, 0.904. The higher purity is due to the decrease in the absorbance of the total protein at 280 nm while the absorbance of the specific phycocyanin at 620 nm increased. The phycocyanin isolated using the high-pressure process is thought to be stable because it was isolated under low-temperature and high-pressure conditions; these conditions destroyed the cell membrane of *Spirulina platensis* without denaturing phycocyanin, which is a chromoprotein that is composed of temperature-sensitive α and β polypeptide subunits [13]. The hexane separation method can be used as a new separation method because it requires fewer chemical components, a simpler separation process, and a shorter separation time. Thus, the high-pressure process with the hexane separation method used in this study is considered to be the best method to isolate under stable coditions a highly purified phycocyanin and is expected to solve the issues associated with the current method, such as the denaturation and destruction of phycocyanin, the long separation time, and the high cost.

Thus, if the phycocyanin is used in the cosmetic industry in the future, the hexane separation method is expected to establish itself as a more effective and stable method due to its use of fewer chemical components and its shorter and simpler process.

### 2.3. SDS-PAGE Analysis of Phycocyanin

**Figure 1:**
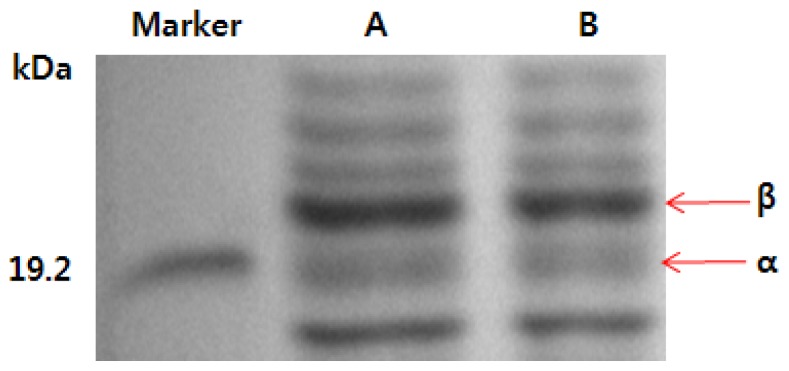
SDS-PAGE of phycocyanin. (**A**) phycocyanin isolated using the hexane separation method and high-pressure process; (**B**) phycocyanin standard (a commercial product).

The molecular weights of the phycocyanin isolated using the high-pressure process and hexane separation method described in this study and those of the phycocyanin standard are shown in Figure 1. The α and β polypeptide subunits of the phycocyanin standard were detected, and their molecular weights were 18.4 kDa and 21.3 kDa, respectively. These weights are consistent with those of previous reports [14]. Moreover, the phycocyanin isolated using the high-pressure process and hexane separation method had two α and β polypeptide subunits that migrated to the same location on the gel and had the same molecular weights as the phycocyanin standard. Furthermore, the bands were clearer than those of the phycocyanin standard. This finding suggests that the high-pressure process and hexane separation method enabledd isolation of a stable, highly purified phycocyanin with a short treatment time using fewer chemical components and increasing the structural stability of the α and β polypeptide subunits.

### 2.4. Stability of Phycocyanin Extract

**Figure 2:**
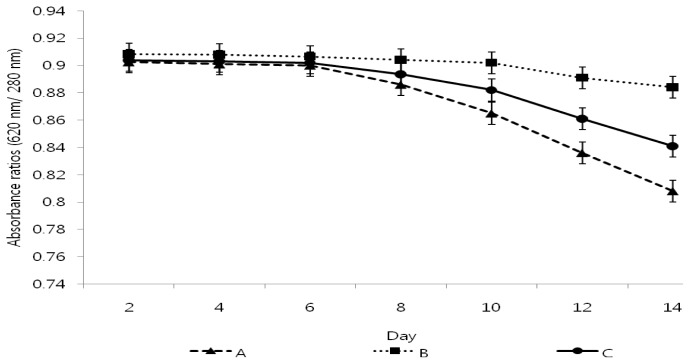
The absorbance ratios of phycocyanin isolated from two different extraction processes such as a conventional hexane separation method and high-pressure process over time. (**A**) phycocyanin isolated using the conventional separation method; (**B**) phycocyanin isolated using the hexane separation method and high-pressure process; (**C**) phycocyanin standard (a commercial product).

To measure the stability of the phycocyanin isolated using the high-pressure process and hexane separation method, the changes of the absorbance of the phycocyanin solution were measured over time, as shown in Figure 2. The phycocyanin from the conventional extraction process had the poorest stability because the absorbance of the phycocyanin from the conventional separation method decreased to 0.808 after 14 days while the phycocyanin standard decreased to 0.841 after 14 days. However, the absorbance of the phycocyanin isolated using the high-pressure process and hexane separation method remained over 0.884 for 14 days. These data suggest that the phycocyanin isolated using the high-pressure process and hexane separation method, compared to two other samples, was more stable because the low temperature and high pressure extraction process effectively destroyed the cell membrane of *Spirulina platensis* while minimizing the damage to the polypeptide subunits of phycocyanin and increasing the elution of phycobiliprotein. Furthermore, the reduced chemical usage, simple separation process, and short process time of the hexane separation method contributed to the attainment of stable phycocyanin containing two chromophore subunit proteins intact along with a relatively pure form. Phycocyanin with high stability and purity is expected to have an application as a new material in the cosmetic and beauty industry.

### 2.5. Measurement of DPPH (α,α-diphenyl-β-picrylhydrazyl) Scavenging Activity

**Figure 3:**
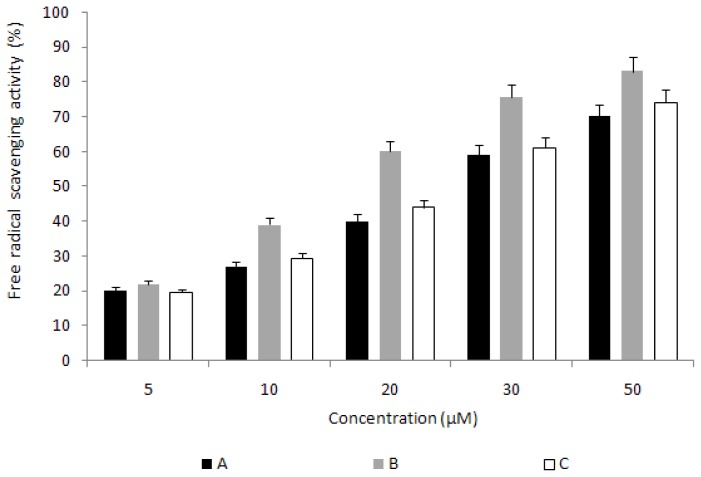
Comparison of free radical scavenging activity of phycocyanins from two different extraction processes. (**A**) phycocyanin isolated using the conventional separation method; (**B**) phycocyanin isolated using the hexane separation method and high-pressure process; (**C**) phycocyanin standard (a commercial product).

To verify the antioxidant activity of phycocyanin, the electron donating ability of the phycocyanin standard and of the phycocyanin isolated using the high-pressure process and hexane separation method were measured. The results are shown in Figure 3. All of the phycocyanins showed increased activity as their concentrations were increased. Phycocyanin isolated using the high-pressure process and hexane separation method showed a high removal rate of oxygen free radicals (83%) at a concentration 50 μM and showed 9% higher activity than that of the phycocyanin standard. These data imply that the purified sample from our process contained more stable chromophore phycocyanin than the samples from conventional processes, which results in better anti oxidant activity than the others.

### 2.6. Measurement of Reducing Power

Reducing power is defined as the ability to donate electrons to free oxygen radicals and other free radicals and is one of the various mechanisms of antioxidant activities; thus, a measurement of reducing power can be used to verify antioxidant activity. All of the phycocyanins showed increased reducing power with increased concentration. Moreover, in the reducing power test, phycocyanin isolated using the described method showed a peak absorbance value of 0.38 at a concentration of 50 μM, which was 0.1 higher than the absorbance of the phycocyanin standard and phycocyanin isolated using the conventional separation method (Table 3). This result, along with the measurement of DPPH scavenging activity, implies that the isolation of stable and highly purified phycocyanin with enhanced antioxidant activity is possible with the high-pressure process and hexane separation method. Furthermore, phycocyanin could be used as a material with antioxidant and anti-aging activities in the cosmetic and beauty industry.

## 3. Experimental Section

### 3.1. Extraction from *Spirulina platensis*

In this study, *S. platensis* from a culture aquarium was filtered using a filter membrane and washed with water to prepare a dried powder. Washed *S. platensis* was powdered using a freeze-dryer, and it was used in the experiment after grinding in a grinder. Ten volumes of distilled water were added to 100 g of *S. platensis* powder and sealed tightly in a special plastic bag, and then hydrostatically pressurized in a 1 L volume high-pressure extraction vessel (Ilshin autoclave, Daejeon, Korea) at 5000 bar and 20 °C for 15 min [15]. After the high-pressure treatment, the sample was immersed in water at 4 °C for 12 h, and filtered by vacuum filtration using filter paper. Then, it was incubated at 4 °C for 12 h, and the water layer was isolated using fractionation. The phycocyanin in the water layer was collected and powdered by freeze-drying as a sample.

### 3.2. Isolation of Phycocyanin

To remove beta-carotene and chlorophyll, which have low light stability, and to isolate phycocyanin from the high pressurized sample, the *Spirulina platensis* extract from the high-pressure treatment was mixed with hexane (Sigma, St. Louis, MO, USA) in a 1:1 volume ratio and shaken using a vortex mixer (VM-96B, JEIO TECH, Daejeon, Korea) for 5 min. Then, it was incubated at 4 °C for 12 h, and the water layer was isolated using fractionation. The phycocyanin in the water layer was collected and powdered by freeze-drying as a sample.

For comparison with the separation method of this study, phycocyanin powder was also extracted by a conventional separation method: In the first, approximately 5.0 g of *S. platensis* was suspended in 200 mL of 0.1 M Na-phosphate buffer pH 7.0 containing 100 g/mL lyzozyme and 10 mM EDTA. The enzymatic disintegration of the cell-wall was brought about by placing the *S. platensis* powder in a shaking bath at 30 °C for 24 h. The slurry was then centrifuged for 1 h at 40,000 × *g* to remove cell debris, yielding a clear supernatant. In the second, the crude phycocyanin was precipitated in 50% ammonium sulfate and was then recovered by centrifugation at 10,000× *g* for 10 min. The colourless, clear supernatant was discarded and the blue precipitate was dissolved in a small volume of 0.0025 M Na-phosphate buffer pH 7.0 and dialyzed against the same buffer. The dialyzed phycocyanin was then placed in a 2.5 × 30 cm hydroxylapatite column and the two main fractions were pooled following a stepwise elution with phosphate buffers of increasing ionic strength at pH 7.0, as follows: The first fraction was eluted between 2.5 mM and 70 mM and represented c-phycocyanin. The fraction containing c-phycocyanin was purified by chromatography on a 1.5 × 20 cm DEAE Sephadex A-50, according to Binder *et al.* [16,17].

### 3.3. Measurement of Extraction Yield and the Purity of Phycocyanin

The extraction yield of the phycocyanin from the three different methods was estimated by the procedure below. The phycocyanin extract in the above method was prepared using three different methods:by the hexane separation method and the high-pressure process used in this study, by the hexane separation method only without the high-pressure process, and by the conventional separation method.

(1) Weight of phycocyanin extract (g)/Weight of spirulina platensis powder (g)×100%

The purity of isolated phycocyanin was checked by the procedure below by the ratio of 280 nm for absorbance of total proteins in phycocyanin to 620 nm of specific protein in the complex [18].

(2) $$\text{PP }(\text{phycocyanin purity})={\text{OD}}_{620\;\text{nm}}/{\text{OD}}_{280\;\text{nm}}$$

The purity of the standard, a commercially available phycocyanin (Anaspec, Inc., CA, USA) was also measured by the above method [18].

### 3.4. SDS-PAGE Pattern of Phycocyanin

In addition to the measurement of purity, for a closer examination of the phycocyanin isolated using the high-pressure process and the hexane separation method, the molecular weight was confirmed using sodium dodecyl sulfate-polyacrylamide gel electrophoresis (SDS-PAGE, Mini-Protean, Bio-rad Ltd., Hercules, CA, USA) and compared with a phycocyanin standard (Anaspec, Inc., CA, USA). SDS-PAGE was performed using a 12% polyacrylamide slab gel and a 4.5% stacking gel and was confirmed after staining with Coomassie blue R25 (Sigma, St. Louis, MO, USA) and destaining. The molecular weights of the phycocyanin subunits were calculated by comparison with a standard ladder (pre-stained SDS-PAGE standard; Bio-Rad, Seoul, Korea) [14].

### 3.5. Stability of Phycocyanin

To measure the stability of the phycocyanin pigment isolated from *Spirulina platensis* at a given temperature, an aqueous solution of phycocyanin was incubated at 40 °C under UV exposure. Then, changes in phycocyanin purity were calculated from the absorbance values at 620 nm and 280 nm with an ELISA reader [19].

### 3.6. Measurement of DPPH (α,α-diphenyl-β-picrylhydrazyl) Scavenging Activity

DPPH scavenging activity was measured using Kang’s method [20]. One milliliter of a 200-μM DPPH solution was added to a series of phycocyanin standards made by solubilizing in ethanol and 2 mL of the phycocyanin isolated using the high-pressure process and hexane separation method. The mixture was vortexed for 10 s to mix completely and incubated at room temperature for 30 min. To measure the remaining DPPH, the absorbance was measured at 517 nm by a UV-vis spectrophotometer (852A Diode Array Spectrophotometer, Agilent Technologies, Seoul, Korea).

### 3.7. Measurement of Reducing Power

The reducing power was measured as follows: 2.5 mL of 200 mM sodium phosphate buffer (pH 6.6) and 2.5 mL of 1% potassium ferricyanide were added to a series of phycocyanin standard concentrations and 2.5 mL of the phycocyanin isolated using the high-pressure process and hexane separation method. The mixture was incubated at 50 °C for 20 min. Then, 2.5 mL of trichloroacetic acid (10%, *w*/*v*) was added and centrifuged at 650× *g* for 10 min. Five milliliters of centrifuged supernatant was mixed with 5 mL of deionized water and 1 mL of 1% ferric chloride, and its absorbance was measured at 700 nm using a UV-vis Spectrophotometer (852A Diode Array Spectrophotometer, Agilent Technologies, Seoul, Korea) [21].

### 3.8. Statistics

All of the analyzed values were presented as the mean ± S.D., and all of the experimental data were analyzed using the statistics software SPSS 16.0. Tests of the significance of differences were analyzed by ANOVA (analysis of variance) and DMRT (Duncan’s multiple range test) at the level of α = 0.05.

## 4. Conclusions

In this study, a high-pressure extraction process associated with hexane obtained relatively stable phycocyanin from *S. platensis* as well as in high purity. This process also has the advantage of using fewer steps and having a shorter process time, which may have contributed to the improvement in the stability and the purity of the phycocyanin [18]. Therefore, these results suggest application of this combined process for expanding the uses of phycocyanin in the cosmetic and food industries, *etc*.

## Figures and Tables

**Table 1:** Yields of phycocyanin isolated from *Spirulina.*

Sample	Yields (%)
A	7.2 ± 0.5
B	10.2 ± 0.2
C	8.4 ± 0.4

A: phycocyanin isolated using the conventional separation method; B: phycocyanin isolated using the hexane separation method and high-pressure process; C: phycocyanin isolated using the hexane separation method.

**Table 2:** Absorbance ratios of phycocyanin isolated from *Spirulina*.

Sample	Absorbance ratios (nm)		

	280	620	620/280
A	2.114 ± 0.005	1.868 ± 0.012	0.884 ± 0.004
B	2.104 ± 0.007	1.912 ± 0.004	0.909 ± 0.003
C	2.125 ± 0.008	1.873 ± 0.009	0.882 ± 0.006
D	2.105 ± 0.006	1.903 ± 0.004	0.904 ± 0.005

A: phycocyanin isolated using the conventional separation method; B: phycocyanin isolated using the hexane separation method and high-pressure process; C: phycocyanin isolated using the hexane separation method. D: phycocyanin standard (a commercial product).

**Table 3:** Comparison of reducing power of phycocyanins from two different extraction processes.

	Reducing power (O.D.)				
	
Sample	Concentration (μM)				
	
	5	10	20	30	50
A	0.07 ± 0.13	0.15 ± 0.12	0.15 ± 0.08	0.18 ± 0.04	0.19 ± 0.06
B	0.08 ± 0.04	0.16 ± 0.09	0.25 ± 0.13	0.31 ± 0.06	0.38 ± 0.05
C	0.09 ± 0.04	0.15 ± 0.18	0.18 ± 0.04	0.20 ± 0.06	0.21 ± 0.08

A: phycocyanin isolated using the conventional separation method; B: phycocyanin isolated using the hexane separation method and high-pressure process; C: phycocyanin standard (a commercial product).
